# Contribution of Risk Factors to Extremely, Very and Moderately Preterm Births – Register-Based Analysis of 1,390,742 Singleton Births

**DOI:** 10.1371/journal.pone.0060660

**Published:** 2013-04-05

**Authors:** Sari Räisänen, Mika Gissler, Juho Saari, Michael Kramer, Seppo Heinonen

**Affiliations:** 1 Department of Obstetrics and Gynaecology, Kuopio University Hospital, Kuopio, Finland; 2 National Institute for Health and Welfare (THL), Helsinki, Finland; 3 Nordic School of Public Health, Gothenburg, Sweden; 4 Kuopio Welfare Research Centre (KWRC), Department of Social Sciences, The University of Eastern Finland, Kuopio, Finland; 5 Department of Epidemiology, Rollins School of Public Health, Emory University, Atlanta, Georgia, United States of America; Tabriz University of Medical Sciences, Iran (Republic of Islamic)

## Abstract

**Background:**

Preterm birth, defined as birth occurring before 37 weeks gestation, is one of the most significant contributors to neonatal mortality and morbidity, with long-term adverse consequences for health, and cognitive outcome.

**Objective:**

The aim of the present study was to identify risk factors of preterm birth (≤36+6 weeks gestation) among singleton births and to quantify the contribution of risk factors to socioeconomic disparities in preterm birth.

**Methods:**

A retrospective population–based case-control study using data derived from the Finnish Medical Birth Register. A total population of singleton births in Finland from 1987−2010 (n = 1,390,742) was reviewed.

**Results:**

Among all singleton births (*n* = 1,390,742), 4.6% (*n* = 63,340) were preterm (<37 weeks), of which 0.3% (*n* = 4,452) were classed as extremely preterm, 0.4% (*n* = 6,213) very preterm and 3.8% (*n* = 54,177) moderately preterm. Smoking alone explained up to 33% of the variation in extremely, very and moderately preterm birth incidence between high and the low socioeconomic status (SES) groups. Reproductive risk factors (placental abruption, placenta previa, major congenital anomaly, amniocentesis, chorionic villus biopsy, anemia, stillbirth, small for gestational age (SGA) and fetal sex) altogether explained 7.7−25.0% of the variation in preterm birth between SES groups.

**Conclusions:**

Smoking explained about one third of the variation in preterm birth groups between SES groups whereas the contribution of reproductive risk factors including placental abruption, placenta previa, major congenital anomaly, amniocentesis, chorionic villus biopsy, anemia, stillbirth, SGA and fetal sex was up to one fourth.

## Introduction

Preterm birth, defined as birth occurring before 37 weeks gestation, is one of the most significant contributors to neonatal mortality and morbidity, with long-term adverse consequences for health, and cognitive outcome as well as financial implications for health care [1]–[3]. Worldwide, approximately 9.6% of all births are preterm, but incidences vary by time and region. The highest rates of preterm births have been observed in developing countries [1], whilst rates from 5% to 12% were reported for Europe in 2004 [4]. Preterm birth rates have been shown to be rising in some developed countries [5], [6]. However, in Finland, a decreasing trend has been reported for the years 1987−2005 [7]. The etiology of preterm births is not completely understood, but it is known to be multifactorial. Currently, there are no effective diagnostic measures for preterm birth [1], but several reproductive risk factors have been identified, such as prior spontaneous preterm birth [8], multiple pregnancies [9], primiparity [7], advanced maternal age [7], [10], [11], smoking [7], [9], obesity [12], preeclampsia [10], genital infections [10], congenital anomalies [13] and assisted reproductive technology (ART) [7]. Further, several previous studies have found significant socioeconomic differences in preterm births, e.g., the risk of prematurity has been shown to be associated with occupation and educational level [11], [14]–[17].

The aim of the present study was to identify risk factors of singleton preterm birth (<37 weeks gestation) and to quantify the contribution of the risk factors to the observed socioeconomic gradient or disparity in preterm births using the total population of singleton births (n = 1,390,742) for the years 1987–2010 in Finland with around 5.5 million residents and mainly publicly funded health services.

## Methods

The source of data used in this study was the Finnish Medical Birth Register (MBR), which is a compilation of the clinical records from all the obstetric care units in Finland. The data for the years 1987 to 2010 were obtained from the National Institute for Health and Welfare (THL), the current register keeper. The MBR includes information on maternal and neonatal birth characteristics and perinatal outcomes covering all live births or stillbirths delivered after the 22nd gestational week or weighing 500 g or more collected from all delivery units in Finland. For each infant, an electronic form, or rarely a paper form, has to be completed by the hospital covering the first seven postnatal days. The reporting units and register controller actively collaborate to guarantee the validity of the data. For example, information submitted to the MBR is checked and missing or apparently, incorrect information is confirmed by contacting the treating hospitals before correcting. Less than 0.1% of newborns are missing from the MBR, and therefore it was supplemented with data compiled by the Population Register Centre on live births and data compiled by Statistics Finland on stillbirths and deaths during the first week of life. After these additions, the data covered 100% of birth events.

Preterm birth was defined as birth before or at 36+6 gestational weeks, being further divided by gestational age to moderately preterm (32+0–36+6), very preterm (28+0–31+6) and extremely preterm (≤27+6). Estimation of the gestational age was based on data for the last menstrual period, unless there was a discrepancy of more than seven or 14 days at the first- or second-trimester ultrasonography measurements, respectively. Information on gestational age was missing in 8,754 (0.6%) cases, which were therefore excluded from the data. Information on in-vitro fertilization (IVF) that included also intracellular sperm injection (ICSI) and frozen embryo transfers (FET) was only available from 1991 onwards. However, only 170 IVF children were born in Finland prior to 1991. Maternal smoking was recorded as either non-smoking or smoking, and after 1991, information on women who had quit smoking during the first trimester was also used. Data on maternal height and weight were available from 2004, and body mass index (BMI; body weight in kilograms by the squared height in meters (kg/m2)) was adjusted only for the years 2004−2010. Small for gestational age (SGA) was defined as fetal growth more than 2 SDs below the sex- and gestational age-specific reference mean (Unpublished MBR data). Socioeconomic status (SES) groups were based on maternal occupation at the time of birth and categorized as upper white-collar workers, lower white-collar workers, blue-collar workers, and others as described elsewhere [15]. Others included entrepreneurs, students, retired, unemployed, housewives and unclassifiable cases. Information on SES groups has been gathered in the MBR since October 1990; 388,469 (27.5%) missing cases were analyzed as separate group in the multivariate analyses. Parturients’ place of residence was grouped by 20 hospital districts in Finland. Anemia was defined as hemoglobin levels below 100 g/L. Marital status was recorded as married, cohabiting or single. The study period 1987−2010 was divided into five time periods (1987−1991, 1992−1996, 1997−2001, 2002−2006, 2007−2010). Apart from birthweight, the number of deliveries, miscarriages and prior terminations, all variables were dichotomous or categorical.

Information on congenital anomalies was taken from the national Congenital Malformations Register established in 1963 and currently maintained by the THL. The register includes data on major congenital anomalies. The two data sources were linked together using encrypted unique personal identification numbers.

### Participants

The data included all singleton births (n = 1,390,742) for the years 1987–2010 in Finland.

### Ethics

The permission to use the confidential register data in this study was approved February 16th in 2012 by the National Institute for Health and Welfare (THL) in Finland. (Reference number 1749/5.05.00/2011). We used only anonymized data, and thus informed consent of the registered individuals was not needed.

### Statistical Methods

Statistical differences in frequencies (categorical and dichotomous variables) between the preterm birth groups were evaluated by the Chi Square test. Differences between continuous variables were evaluated by Student’s t and Mann Whitney’s U tests as appropriate. Multivariable logistic regression analyses were used to model the risk factors separately for each preterm birth group (extremely preterm, very preterm and moderately preterm). In the multivariable analyses, the reference population included women who delivered at term (gestational weeks 37 or more). Possible independent variables were selected based on bivariate analyses (p<0.05). Differences were deemed to be significant if p<0.05. 95% confidence intervals (CI) were also calculated.

Furthermore, to examine the contribution of reproductive risk factors (smoking, placental abruption, placenta previa, major congenital anomaly, anemia, stillbirth, SGA and fetal sex) and obstetric interventions (amniocentesis, chorionic villus biopsy and IVF) to preterm birth incidence between SES groups, we measured the percentage reductions for each preterm birth group by using logistic regression. Reproductive risk factors and obstetric interventions were added separately to model 3 (adjusted by SES, maternal age, parity and smoking), and the percentage reduction in the odds ratio (OR) of each preterm birth group was measured. The formula used was: (OR Model 2– OR Model ×)/(OR Model 2–1) [18]. The data were analyzed using SPSS for Windows 19.0, Chicago, IL.

## Results

The study population corresponded to all singleton births (n = 1,390,742) in Finland from 1987–2010, of which 4.6% (n = 64,843) were preterm (≤36+6 weeks). Of all the preterm births, 0.3% (n = 4,452) were classed as extremely preterm, 0.4% (n = 6,213) were very preterm and 3.9% (n = 54,177) moderately preterm, as shown in Table 1. The rate of preterm births decreased from 4.7% in 1997−2001 to 4.5% in 2007−2010. Women who delivered preterm were significantly more often primiparae, of advanced age (≥30 years), obese (BMI≥30) and smokers compared with women who delivered at term. Women in the study groups more often exhibited reproductive risk factors, such as placenta previa and placental abruption, than the reference population. Socioeconomic differences were also observed. All deliveries were grouped by parturients’ place of residence and hospital districts. The incidence of extremely preterm births varied significantly from 0.27% to 0.38%, very preterm births from 0.34% to 0.53% and moderately preterm births from 3.31% to 4.70% among the 20 hospital districts in Finland (p≤0.001), but we were not able to observe any clear geographical patterns.

**Table 1:** Demographic characteristics and reproductive risk factors among each singleton preterm birth group and term births (*n* = 1,390,742) for the years 1987–2010 in Finland.

Characteristic	Extremely preterm	Very preterm	Moderately preterm	Term	*p* value
	(<28 weeks)	(28–31+6 weeks)	(32–36+6 weeks)	(≥37 weeks)	
*n* (%)	4452 (0.3%)	6213 (0.4%)	54,177 (3.8%)	1,338,438 (99.4%)	
Mean maternal age (±SD) (years)	30.1 (±6.0)	29.8 (±5.9)	29.4 (±5.7)	29.1 (±5.3)	≤0.001
Primiparity	43.2	48.3	49.0	40.4	≤0.001
Pregravid BMI^a^	25.0 (±5.7)	24.8 (±5.3)	24.4 (±5.1)	24.2 (±4.7)	≤0.001
Non-smoking	78.3	78.2	81.4	84.7	≤0.001
Quitted smoking	3.8	4.2	3.7	3.5	
Smoking	18.0	17.7	14.9	11.8	
Married or cohabiting	94.1	93.6	94.5	95.9	≤0.001
Socioeconomic status^b^					
Upper white-collar worker	5.2	5.6	6.1	6.6	≤0.001
Lower white-collar worker	29.3	31.2	32.3	32.6	
Blue-collar worker	14.3	14.6	14.4	13.7	
Other	18.8	18.9	19.4	19.9	
Missing	32.4	29.8	27.8	27.2	
Mean number of deliveries (±SD)	0.57±0.50	0.52±0.50	0.51±0.50	0.60±0.50	≤0.001
Mean number of miscarriages (±SD)	0.50±0.99	0.39±0.81	0.31±0.72	0.26±0.61	≤0.001
Mean number of prior terminations (±SD)	0.19±0.56	0.17±0.53	0.14±0.45	0.12±0.41	≤0.001
IVF^b^	1.5	1.3	1.3	0.7	≤0.001
Anemia ≤100 g/l	1.1	0.8	0.6	0.6	≤0.001
Chorionic villus biopsy	2.0	1.5	1.1	0.9	≤0.001
Amniocentesis	6.5	5.2	4.2	2.7	≤0.001
Placenta previa	1.1	1.9	1.6	0.1	≤0.001
Placental abruption	3.5	4.4	1.6	0.1	≤0.001
Congenital anomalies	11.5	14.0	7.4	2.7	≤0.001
Induction	15.3	8.5	12.6	13.5	≤0.001
Cesarean section	28.6	56.1	31.4	14.3	≤0.001
Stillbirth	4.1	2.8	1.6	0.8	≤0.001
Boy	54.2	55.8	55.8	50.9	≤0.001
Mean birthweight (±SD) (grams)	752.6±350.3	1420.2±454.2	2643.1±580.3	3607.5±478.4	≤0.001
SGA (<−2 SDs below the mean)	19.6	20.6	10.3	2.7	≤0.001
Time periods					
1987–1991	0.4	0.5	3.8	95.3	≤0.001
1992–1996	0.3	0.4	3.8	95.4	
1997–2001	0.3	0.5	4.0	95.3	
2002–2006	0.3	0.4	3.9	95.4	
2007–2010	0.3	0.4	3.8	95.5	
Region (20) range	0.27−0.38	0.34−0.53	3.31−4.70	94.45−95.97	

SD = standard deviation, IVF = in vitro fertilization,

a BMI = body mass index, corresponding to the years 2004–2010, *n* = 369,546,

b corresponding to the years 1991–2010.

The risk factors of preterm birth appeared to fall into two groups: those contributing to the incidence over the entire range of preterm birth and those contributing to the incidence of only the most extreme class preterm births. Risk factors placing women at a high risk (adjusted odds ratio (aOR) >2.0) of extremely, very and moderately preterm births were SGA, placenta previa, placenta abruption, stillbirth and major congenital anomaly, whereas IVF, amniocentesis and anemia were only associated with a high risk of extremely preterm birth (Table 2).

**Table 2:** Adjusted odds ratios (aORs) of singleton extremely preterm, very preterm and moderately preterm births for the years 1987–2010 in Finland.

Characteristic/risk factor	aExtremely preterm	aVery preterm	aModerately preterm
	(<28 weeks), *n* = 3,079	(28–31+6 weeks), *n* = 4,757	(32–36+6 weeks), *n* = 44,390
Maternal age (year)			
≤19	1.32 (1.06–1.63)*	1.24 (1.05–1.46)*	1.07 (1.01−1.14)*
20−29	1	1	1
30−39	1.25 (1.16–1.36)***	1.24 (1.17–1.33)***	1.13 (1.10–1.15)***
≥40	1.49 (1.26–1.76)***	1.60 (1.38–1.85)***	1.48 (1.40–1.56)***
Primiparity	1.31 (1.12–1.54)***	1.41 (1.33–1.50)***	1.49 (1.46–1.52)***
Pregravid BMI^b^			
≤24.9	1	1	1
25−29.9	1.10 (0.92–1.32)	1.21 (1.05–1.40)**	1.00 (0.95−1.04)
≥30	1.48 (1.20–1.82)***	1.46 (1.24–1.74)***	1.20 (1.14–1.27)***
Non-smoking	1	1	1
Quitted smoking	0.98 (0.80–1.19)	1.18 (1.02–1.36)**	1.00 (0.95–1.05)
Smoking	1.21(1.09–1.34)***	1.23 (1.33–1.34)***	1.15 (1.11–1.18)***
Not married or cohabiting	1.31 (1.12–1.54)***	1.32 (1.16–1.50)***	1.20 (1.15–1.25)***
Socioeconomic position			
Upper white-collar worker	1	1	1
Lower white-collar worker	1.23 (1.04–1.46)*	1.15 (1.01–1.31)*	1.09 (1.05–1.14)***
Blue-collar worker	1.30 (1.08–1.57)**	1.14 (0.99–1.32)	1.11 (1.06–1.17)***
Other	1.27 (1.07–1.52)**	1.11 (0.96–1.26)	1.07 (1.02–1.12)***
Missing	1.49 (1.24−1.79)***	1.20 (1.04–1.38)**	1.12 (1.07–1.17)***
Prior miscarriages	1.41 (1.36–1.46)***	1.26 (1.22–1.31)***	1.14 (1.12–1.16)***
Prior terminations	1.28 (1.20–1.37)***	1.16 (1.09–1.23)***	1.07 (1.05–1.10)***
IVF^c^	2.14 (1.63–2.82)***	1.52 (1.18–1.96)***	1.49 (1.37–1.62)***
Anemia ≤100 g/l	2.48 (1.82–3.38)***	1.48 (1.08–2.04)*	0.99 (0.88–1.12)
Chorionic villus biopsy	1.80 (1.38–2.33)***	1.20 (0.93–1.56)	1.04 (0.94–1.15)
Amniocentesis	2.04 (1.75–2.37)***	1.58 (1.38–1.82)***	1.34 (1.27–1.41)***
Placenta previa	6.31 (4.52–8.80)***	10.00 (7.90–12.65)***	10.03 (9.14–11.00)***
Placental abruption	23.41 (18.87–29.04)***	31.69 (29.92–37.32)***	12.18 (11.04–13.44)***
Major congenital anomaly	3.57 (3.17–4.01)***	4.70 (4.31–5.12)***	2.51 (2.41–2.61)***
Induction	1.03 (0.93−1.13)	0.49 (0.44−0.54)***	0.87 (0.85−0.90)***
Stillbirth	4.46 (3.68−5.42)***	3.73 (3.10−4.49)***	2.28 (2.10−2.47)***
SGA	7.35 (6.69−8.09)***	7.93 (7.35−8.55)***	3.65 (3.53−3.78)***
Boy	1.13 (1.06–1.22)***	1.20 (1.13–1.27)***	1.21 (1.19–1.24)***
Time periods			
1987−1991	1.47 (1.29–1.67)***	1.38 (1.24–1.53)***	1.10 (1.06–1. 14)***
1992−1996	1.42 (1.26–1.60)***	1.28 (1.16–1.42)***	1.07 (1.04–1.11)***
1997−2001	1.28 (1.14–1.44)***	1.28 (1.16–1.42)***	1.07 (1.03–1.10)***
2002−2006	1.10 (0.97–1.24)	1.14 (1.03–1.26)**	1.03 (1.00–1.07)*
2007−2010	1	1	1

a Reference population: singleton term pregnancies (≥37 weeks), *n* = 1,133,367,

b BMI = body mass index, corresponding to the years 2004−2010, reference group *n* = 236,091.

c IVF = in-vitro fertilization, corresponding to the years 1991−2010.

Risk factors placing women at a moderate risk (aOR<2.0) of extremely, very and moderately preterm births were advanced maternal age, primiparity, obesity (BMI≥30), smoking, being single, of low socioeconomic status, prior miscarriages, prior terminations and male fetal sex (Table 2). Furthermore, IVF and amniocentesis were associated with a moderate risk of very and moderately preterm birth and chorionic villus biopsy with a moderate risk of extremely preterm birth.

The contribution of reproductive risk factors of preterm birth and obstetric interventions to socioeconomic disparities in preterm birth were studied by using logistic regression and comparing the percentage reductions in the ORs, as shown in Table 3. Only contributions above zero were considered. Model 1 considered the OR of each preterm birth group adjusted by SES alone. After smoking was added to Model 2 (adjusted for SES, age and parity), the OR, representing the gap between preterm birth odds in high as compared to low SES women, decreased and smoking alone explained 10.3−26.2%, 16.7−33.3% and 20.0−29.6% of the variation in extremely, very and moderately preterm incidence between the SES groups, respectively. After reproductive risk factors were added to Model 3, the ORs of extremely preterm births decreased, and reproductive risk factors explained altogether 7.7−25.0% of the difference in extremely, very and moderately preterm incidence between the SES groups. The contribution of obstetric interventions to preterm birth incidence appeared to be null with the exception that they explained 3.8% of variation in extremely preterm birth between upper and lower white-collar workers (Model 5). Further, maternal age and parity did not contribute to the variation in preterm birth incidence between the SES groups.

**Table 3:** ORs of extremely preterm, very preterm and moderately preterm births after adjustments for characteristics and risk factors.

	Model 1	Model 2	Model 3		Model 4		Model 5	
	OR (95% CI)	OR (95% CI)	OR (95% CI)	Diff.with 2 (%)*	OR (95% CI)	Diff. with 3 (%)*	OR (95% CI)	Diff. with 3 (%)*
**Extremely preterm birth**								
Upper white-collar	1	1	1		1		1	
Lower white-collar	1.18 (1.02−1.36)	1.29 (1.12−1.49)	1.26 (1.08−1.46)	10.3	1.24 (1.06−1.44)	7.7	1.25 (1.08−1.46)	3.8
Blue-collar worker	1.38 (1.18−1.61)	1.61 (1.38−1.89)	1.45 (1.23−1.71)	26.2	1.37 (1.16−1.62)	17.8	1.45 (1.23−1.71)	–
Other	1.23 (1.06−1.43)	1.41 (1.21−1.64)	1.37 (1.17−1.60)	9.8	1.32 (1.13−1.56)	13.5	1.37 (1.17−1.60)	–
Missing	1.67 (1.45−1.93)	1.76 (1.53−2.03)	1.67 (1.44−1.94)	11.8	1.67 (1.43−1.95)	–	1.72 (1.48−2.00)	–
**Very preterm birth**								
Upper white-collar	1	1	1		1		1	
Lower white-collar	1.14 (1.02−1.28)	1.24 (1.10−1.39)	1.20 (1.06−1.35)	16.7	1.18 (1.04−1.33)	10.0	1.20 (1.06−1.35)	–
Blue-collar worker	1.28 (1.13−1.45)	1.48 (1.31−1.68)	1.32 (1.16−1.50)	33.3	1.24 (1.09−1.42)	25.0	1.32 (1.16−1.50)	–
Other	1.13 (1.00−1.27)	1.27 (1.13−1.44)	1.19 (1.05−1.35)	29.6	1.15 (1.01−1.30)	21.1	1.19 (1.05−1.35)	–
Missing	1.32 (1.18−1.48)	1.46 (1.30−1.65)	1.39 (1.23−1.56)	15.2	1.41 (1.25−1.59)	–	1.41 (1.25−1.58)	–
**Moderately preterm birth**								
Upper white-collar	1	1	1		1		1	
Lower white-collar	1.09 (1.05−1.13)	1.15 (1.11−1.19)	1.12 (1.08−1.17)	20.0	1.11 (1.06−1.15)	8.3	1.12 (1.08−1.17)	–
Blue-collar worker	1.16 (1.11−1.21)	1.27 (1.22−1.32)	1.19 (1.14−1.24)	29.6	1.16 (1.11−1.21)	15.8	1.19 (1.14−1.24)	–
Other	1.08 (1.03−1.12)	1.16 (1.11−1.20)	1.13 (1.08−1.18)	18.8	1.11 (1.06−1.15)	15.4	1.13 (1.08−1.18)	–
Missing	1.13 (1.09−1.17)	1.21 (1.16−1.25)	1.18 (1.14−1.23)	14.3	1.19 (1.14−1.23)	–	1.19 (1.14−1.24)	–

* (The contribution of each factor was measured by the percentage reduction in the odds ratio of socioeconomic status compared to Model 2 and 3 by using formula (OR Model 2/3– OR Model x)/(OR Model 2/3–1). Model 1 = Adjusted by socioeconomic status. Model 2 = Adjusted by socioeconomic status+age and parity. Model 3 = Adjusted by Model 2+ smoking. Model 4 = Adjusted by Model 3+ placental abruption+placenta previa+major congenital anomaly+anemia+stillbirth+SGA+ fetal sex. Model 5 = Adjusted by Model 3+ amniocentesis+chorionic villus biopsy+IVF.

## Discussion

The aim of the present study was to identify risk factors of singleton preterm births, and to quantify the contribution of risk factors to the socioeconomic gradient in preterm birth incidence using the total population of singleton birth for the years 1987–2010 in Finland. The secular trend of preterm births was decreasing and the total rate of preterm births (4.6%) for the years 1987–2010 was low compared with the rates of 5–7% reported for other developed countries [1]. It might be speculated that improved prenatal diagnosis for elimination of severe congenital anomalies may have an influence on the incidence of preterm but their contribution to preterm birth incidences was minor since the number of pregnancy terminations on fetal indications increased from 188 in 1993 to 339 in 2010 (www.thl.fi). Our data did not include late terminations of pregnancies, since the Finnish ICD-10 classification excludes them from perinatal mortality rates. The main finding of the present study was that smoking alone made the largest contribution and explained in the order of up to 33% of the difference in extremely and moderately preterm incidence and very preterm incidence between SES groups, whereas the contributions of reproductive risk factors (placental abruption, placenta previa, major congenital anomaly, anemia, stillbirth, SGA and fetal sex) explained about 8−25% of the difference in preterm birth incidence between SES groups.

The results of the present study confirmed the multifactorial etiology of prematurity and showed that SES plays a substantial role in preterm births, even after adjustment for numerous birth characteristics and reproductive risk factors. The incidence of preterm births was mostly higher among blue-collar compared to white-collar workers, which may be due to differences in occupational exposures, lifestyle factors and health-promoting behavior between the groups. However, a possible limitation of the study is that the SES groups were based solely on the maternal occupation at time of birth and no information on the father’s occupation was available. Further, cases with missing SES information were close to the general population, not outliers, implying that the bias brought about by the missing data was unlike to affect the results significantly.

Our results also suggested an association between preterm births and several reproductive risk factors, such as primiparity, advanced maternal age, congenital anomalies, IVF, and obesity, which have also been identified in previous studies [7], [10], [13]. However, unlike most previous studies, we quantified the contribution of risk factors to preterm birth incidence. The results showed that the contribution of risk factors varied over the entire range of preterm births and smoking contributed the most, explaining about 10−30% of SES disparities in preterm birth incidence. These figures are in reasonable agreement with a previous study in Finland covering the years 1991 to 1999 [15]. Thus, smoking during pregnancy resulted in 0.2, 0.3 and 5.3 extra cases of extremely, very and moderately preterm births, respectively, per 1,000 births in blue-collar workers compared to upper white-collar workers. However, smoking has been shown to have a dose-dependent relationship to prematurity [19] but since we did not have information on the number of cigarettes smoked per day, we could not examine the dose-dependence. In general, however, smoking habits during pregnancy are relatively well covered in the Finnish MBR [20]. Overall, the contribution of reproductive risk factors appeared to be somewhat lower since placental abruption, placenta previa, major congenital anomalies, anemia, stillbirth, SGA and fetal sex altogether explained up to one fourth of the preterm birth incidences between SES groups (Table 3, Model 4).

A novel finding was that amniocentesis, a common invasive prenatal diagnostic procedure, was associated with preterm births. However, amniocentesis may be a risk factor, suggesting causal association, or a non-causal indicator of high risk pregnancy. Previous results have only identified an association between pregnancy loss and amniocentesis; the total post-amniocentesis pregnancy loss rate was reported to be 1.9% in a previous systematic review [21]. However, contribution of obstetric interventions (amniocentesis, chorionic villus biopsy and IVF) appeared to be null with the exception of less than 5% contribution of differences in extremely preterm birth between two highest SES groups. The observed association between anemia and prematurity agrees with a previous meta-analysis, which reported a slightly increased risk of prematurity in women who suffered anemia in early pregnancy [22]. However, our data did not provide information on the pregnancy stage when anemia was observed. Obesity was associated with a 48% (95% CI 20% to 82%) higher incidence of extremely preterm birth, again in line with previous results [12] but we did not estimate its contribution to the SES disparity of preterm births since the database used only contained information for the years 2004–2010.

### Strengths and Weaknesses

The most important strength of our study was that the data derived from the mandatory, national Finnish Medical Birth Register (MBR) covered the entire population, and therefore offered a comprehensive insight into the trends and risks of preterm births. The content of the MBR is extensive, and thus it offered a unique opportunity to use numerous birth characteristics, perinatal outcomes and socioeconomic factors as exposures. To the best of our knowledge, the present study population is one of the largest studied to date, and the main outcome measure was adjusted for numerous background information such as socioeconomic factors and birth characteristics. Further, unlike previous studies, we quantified the contribution of risk factors to preterm birth incidence. Further, the MBR has been shown to have excellent data coverage and quality [23], [24]. A possible limitation of the study was that gestational age was not estimated by ultrasonography for all women, which may have resulted in obscuring or creating a secular trend in preterm birth rates across the time periods because earlier periods were more reliant on the last menstrual period without correction. Further, it should be noted that we were not allowed to use all possible significant confounding factors found in previous studies such as previous preterm births [8], preeclampsia and chorioamnionitis [10], [25] since the availability and quality of the data in these respects were limited.

We conclude that our most important finding was that smoking as a marker of lifestyle contributed the most, explaining about 10−30% of the differences in extremely, moderately and very preterm birth incidence between SES groups. This is a clearly recognizable risk group, and therefore an ideal target for interventional attempts to reduce the incidence of preterm birth. Further, smoking is a modifiable risk factor, and thus advocating smoking cessation for pregnant women and women attempting to become pregnant seems to be advisable. It is, however, unknown how much of this risk is causal and how much of the risk remains after quitting smoking since the cluster of the risks brought about by lifestyle is probably present without smoking.

Identifying preventable causes of prematurity is one of the most significant challenges in perinatology and health research. Similar to previous studies, the etiology of prematurity appeared to be multifactorial and the risk profile included several reproductive risk factors, lifestyle factors and socioeconomic status. However, up to 78% of the incidence of preterm birth remained unexplained and the reasons were variable, maybe even specific for each case.
